# The Role of Ca^2+^/PI3K/Akt/eNOS/NO Pathway in Astragaloside IV–Induced Inhibition of Endothelial Inflammation Triggered by Angiotensin II

**DOI:** 10.1155/2024/3193950

**Published:** 2024-10-30

**Authors:** Shiyu Zhang, Shijie Li, Shiyang Xie, Lin Cui, Yuan Gao, Youping Wang

**Affiliations:** Division of Cardiology and Central Laboratory, First Affiliated Hospital, Henan University of Traditional Chinese Medicine, Zhengzhou 450000, China

**Keywords:** angiotensin II, astragaloside IV, endothelial cell, endothelial nitric oxide synthase, inflammation, nitric oxide

## Abstract

Inflammation induced by angiotensin II (Ang II) is a key event in the progression of numerous cardiovascular diseases. Astragaloside IV (AS-IV), a glycoside extracted from *Astragalus membranaceus Bunge*, has been shown to inhibit Ang II–induced inflammatory responses in vivo. However, the mechanisms underlying the beneficial effects are still unclear. This study investigated whether AS-IV attenuates endothelial inflammation induced by Ang II via the activation of endothelial nitric oxide synthase (eNOS)/nitric oxide (NO) pathway. Human umbilical vein endothelial cells (HUVECs) were cultured in the presence of AS-IV with or without the specific inhibitor of NOS or Ca^2+^- and phosphatidylinositol 3-kinase (PI3K)/Akt-dependent cascade prior to Ang II exposure. Incubation of HUVECs with AS-IV enhanced NO production and eNOS^ser1177^ phosphorylation. These responses were abrogated by the inhibition of NOS or Ca^2+^- and PI3K/Akt-dependent pathway. In addition, preincubation of HUVECs with AS-IV inhibited Ang II–induced cytokine and chemokine production, adhesion molecule expression, monocyte adhesion, and nuclear factor kappa B (NF-κB) activation as evidenced by the attenuation of inhibitor of kappa B alpha phosphorylation and subsequent NF-κB DNA binding. These effects of AS-IV were abolished by the suppression of NOS or Ca^2+^- and PI3K/Akt-dependent cascade. Our findings indicate that AS-IV attenuates inflammatory responses triggered by Ang II possibly via the activation of Ca^2+^/PI3K/Akt/eNOS/NO pathway in endothelial cells.

## 1. Introduction

Cardiovascular diseases are the major causes of morbidity and mortality worldwide. A growing body of evidence supports a critical role played by endothelial dysfunction, defined as a decrease in nitric oxide (NO) availability, in the pathogenesis of various cardiovascular diseases, including atherosclerosis, hypertension, and heart failure [1–3]. This is due to that endothelial dysfunction promotes vasoconstriction, leukocyte adhesion, and platelet aggregation. Inflammation is one of the significant hallmarks of endothelial dysfunction, which is associated with the release of proinflammatory mediators and leukocyte recruitment [3, 4]. The striking correlation is observed between inflammation and numerous cardiovascular diseases, and the benefits from anti-inflammatory therapies have been shown in several clinical trials [1, 5].

It is well accepted that renin–angiotensin system (RAS) is implicated in the development of various cardiovascular diseases. The overactivation of the RAS usually induces vasopressor and proinflammatory and hypertrophic actions primarily via its principal effector, angiotensin II (Ang II) [6]. Once binding to type-1 angiotensin receptors (AT1R) on endothelial cells, Ang II triggers inflammation and increases the release and expression of proinflammatory cytokines (tumor necrosis factor alpha [TNF-α] and interleukin 6 [IL-6]), chemokines (monocyte chemoattractant protein-1 [MCP-1]), and adhesion molecules (intercellular adhesion molecule 1 [ICAM-1] and vascular cell adhesion molecule 1 [VCAM-1]). Thus, in cardiovascular diseases, triggering the inflammatory responses and increasing the recruitment of leukocytes into the tissues play the major role in Ang II–associated pathogenic effects [6, 7].

NO produced from endothelial nitric oxide synthase (eNOS) is a crucial effective molecule in the regulation of cardiovascular system under physiological states [8]. The physiological effects mediated by NO derived from eNOS are characterized by the maintenance of endothelium-dependent vasorelaxation and inhibition of inflammatory responses and platelet activation. Indeed, there is compelling evidence showing that NO produced from eNOS has anti-inflammatory properties [8, 9]. It is well recognized that the eNOS-induced NO production is regulated by multiple signaling cascades, including Ca^2+^- and phosphatidylinositol 3-kinase (PI3K)/serine/threonine kinase Akt-dependent signaling cascades [9, 10].

Astragaloside IV (AS-IV) is a principal bioactive component extracted from the traditional Chinese medicinal herb *Astragalus membranaceus Bunge* [11], and it has been reported to possess anti-inflammatory and antioxidant properties [11–13]. Several studies have demonstrated that AS-IV protects against Ang II– and diabetes-induced organ damage, including aortic aneurysm and renal injury [14–16]. The protective effects of AS-IV are mainly derived from its anti-inflammatory properties. However, the detailed mechanisms underlying the beneficial effects of AS-IV on Ang II–induced inflammation remain to be fully elucidated. Recently, AS-IV has been found to induce eNOS-mediated NO production in crush syndrome and diabetic nephropathy rats [17, 18]. Although NO derived from eNOS has been demonstrated to inhibit inflammation, no data are available that determine the effects of eNOS-mediated NO production induced by AS-IV on inflammation occurring endothelial cell, especially Ang II–induced inflammation, and its underlying mechanisms.

Therefore, the aim of this study was to investigate the in vitro effects of AS-IV on Ang II–induced inflammation in human umbilical vein endothelial cells (HUVECs) and to identify whether AS-IV inhibits Ang II–induced inflammatory responses of endothelial cells via the activation of Ca^2+^ and PI3K/Akt/eNOS/NO pathway.

## 2. Materials and Methods

### 2.1. Reagents and Chemicals

AS-IV (molecular weight = 784.97 and purity ≥98% by high performance liquid chromatography (HPLC)) was obtained from Shanghai Yuanye Biotechnology Company (Shanghai, China). 3-(4,5-Dimethylthiazol-2-yl)-2,5-diphenyltetrazolium bromide (MTT), dimethyl sulfoxide (DMSO), protease inhibitor solution, sodium nitroprusside (SNP), ethylene glycol tetraacetic acid (EGTA), and Triton X-100 were supplied by Sigma Chemical (St. Louis, MO, USA). Fetal bovine serum (FBS), RPMI-1640 medium, and carboxyfluorescein diacetate succinimidyl ester were purchased from Invitrogen (Carlsbad, CA, USA). N^G^-Monomethyl-L-arginine (L-NMMA) and LY294002 were purchased from Wako Pure Chemicals (Chuo-ku, Osaka, Japan) and Calbiochem (San Diego, CA, USA), respectively. Cytokine and chemokine enzyme-linked immunosorbent assay (ELISA) kits were supplied by R&D Systems (Minneapolis, MN, USA). A protein assay kit was obtained from Bio-Rad Laboratories (Hercules, CA, USA), and radioimmunoprecipitation assay (RIPA) lysis buffer was from Thermo Scientific (Waltham, MA, USA).

### 2.2. Cell Culture and Study Protocol

HUVECs were purchased from Lonza (Allenddale, New Jersey, USA) and maintained in an EGM-2 Bullet kit medium containing growth supplements (Lonza, Allenddale, New Jersey, USA). HUVECs grown to near confluence (80%–90%) were made quiescent by growth supplement deprivation for 4 h prior to the start of experiments. A human monocytic cell line THP-1 from the American Type Culture Collection (ATCC) (Manassas, Virginia, USA) at a density of 2–5 × 10^6^ cells/mL was cultured in RPMI 1640 medium supplemented with 10% FBS, as described in the product sheet provided by the vendor. Cells were cultured at 37°C in a humidified atmosphere containing 5% carbon dioxide (CO_2_) and used between 3 and 7 passages.

To test the impact of AS-IV on NO production and its underlying mechanisms in endothelial cells, 80%–90% confluent HUVECs were preincubated for 1 h with or without the specific inhibitor of NOS (L-NMMA, 1 mmol/L), PI3K/Akt signaling pathway (LY294002, 10 μmol/L), or Ca^2+^-chelating agent (EGTA, 500 nmol/L), followed by incubation with AS-IV (3, 10, or 30 μmol/L) for the indicated times. After then, the media and cells were harvested for the determination of NO production and protein levels of phosphorylated eNOS. In addition, to determine the effect of AS-IV on Ang II–induced inflammation and its possible mechanisms in endothelial cells, confluent HUVECs were preincubated for 1 h with AS-IV (30 μmol/L) or SNP (a NO donor, 30 μmol/L) in the presence or absence of L-NMMA (1 mmol/L), LY294002 (10 μmol/L), or EGTA (500 nmol/L). Then, the cells were incubated with Ang II (1 μmol/L) for the indicated times. After incubation with Ang II, the media and cells were collected and used for further analyses. AS-IV was dissolved in DMSO, and stock solutions (1 mg/mL) were stored at −20°C. The reagents were freshly diluted to the indicated concentrations in the culture medium before the experiment was initiated. As a control, HUVECs were incubated with 0.1% DMSO alone.

### 2.3. Determination of Cell Viability

To determine the cell toxicity of AS-IV, a MTT assay was used to assess the effect of AS-IV on the viability of HUVECs. HUVECs were seeded in 96-well plates to a density of 1 × 10^4^ cells/well 12 h before addition of AS-IV (3, 10, or 30 μmol/L). After 6, 12, or 24 h of incubation, MTT (0.5 mg/mL final concentration) was added to each well and incubated for a further 4 h. After the removal of the media containing MTT, DMSO (100 μL) was added to solubilize the crystals before measurement of absorbance at 570 nm using a SpectraMax microplate reader (Molecular Devices, Sunnyvale, CA, USA). Cell viability is presented as the change relative to the control.

### 2.4. Quantification of NO Production

Since NO is very unstable, the level of nitrite (NO_2_^−^), the stable metabolite of NO, is usually determined to reflect NO production. Confluent HUVECs grown in 24-well plates (0.5 × 10^5^ cells/well) were incubated with various concentrations of AS-IV in the presence or absence of L-NMMA (1 mmol/L), LY294002 (10 μmol/L), or EGTA (500 nmol/L) for the indicated times as described above. Subsequently, the culture medium was collected, and the concentration of nitrite was measured at 540 nm using a nitrate/nitrite colorimetric assay kit (R&D Systems, Minneapolis, MN, USA) following the manufacturer's instruction. The optical density of each sample was assayed at 540 nm using a SpectraMax microplate reader (Molecular Devices, Sunnyvale, CA, USA). Sodium nitrite was used to construct a standard curve. The content of nitrite in each sample was quantified using a sodium nitrite standard curve. Data were expressed as nanomole of the culture media.

### 2.5. Western Blot Analysis

HUVECs grown in 6-well plates (3 × 10^5^ cells/well) were lysed in the ice-cold RIPA containing a protease inhibitor solution (1:10) following incubation with various concentrations of AS-IV in the presence or absence of L-NMMA (1 mmol/L), LY294002 (10 μmol/L), or EGTA (500 nmol/L) for the indicated times as we described earlier, and the supernatants of cell lysates were obtained by centrifugation (12,000 × *g*, 4°C) for 20 min. The concentration of protein in the supernatant was quantified using a Bio-Rad protein assay kit. Equal amounts of protein were separated on 10% sodium dodecyl sulfate–polyacrylamide gels prior to being transferred to a polyvinylidene fluoride (PVDF) membrane. Blots were blocked with 5% skimmed dry milk in Tris-buffered saline with Tween-20 (TBS-T) and then incubated overnight at 4°C with mouse monoclonal anti-phospho-eNOS (Ser1177) (1:800, Invitrogen, Carlsbad, CA, USA) or rabbit monoclonal anti-eNOS (1:1000, Abcam, Cambridge, MA, USA) in blocking solution. After being washed with TBS-T, the blots were incubated with bovine anti-mouse or anti-rabbit horseradish peroxidase-conjugated antibody (1:1000–3000, Santa Cruz Biotechnology, Santa Cruz, CA, USA) at room temperature for 1 h. Finally, the immunoreactive bands were visualized using an enhanced chemiluminescence western blot test kit (Amersham Biosciences, Piscataway, NJ, USA). The densities of bands were quantified using the ImageJ software (National Institute of Health, Bethesda, MD, USA) and normalized to the total amount of eNOS loaded in each well.

### 2.6. Quantification of Cytokine and Chemokine

Confluent HUVECs in 24-well plates (1 × 10^5^ cells/well) were preincubated for 1 h with AS-IV (30 μmol/L) or SNP (30 μmol/L) in the presence or absence of L-NMMA (1 mmol/L), LY294002 (10 μmol/L), or EGTA (500 nmol/L), followed by incubation with Ang II for 6 h. The supernatants were collected by centrifugation and frozen at −80°C until use. The levels of TNF-α, IL-6, and MCP-1 were measured using the corresponding ELISA kits as described by the manufacturer. The levels of TNF-α, IL-6, and MCP-1 were expressed as picogram per milliliter of the culture media.

### 2.7. Cell-Based ELISA Assay

A cell-based ELISA assay was used to determine the effects of AS-IV on the changes in the Ang II–induced expression of adhesion molecules (ICAM-1 and VCAM-1) and phosphorylated inhibitor of kappa B alpha (IκBα) (Ser32) as we reported previously [9]. In brief, HUVECs in 96-well plates were fixed with 4% paraformaldehyde for 5 min following incubation with Ang II for the indicated times and rinsed three times with phosphate-buffered saline (PBS). The fixed cells were permeabilized with prechilled MeOH for 10 min at 4°C prior to being blocked with PBS containing 1% bovine serum albumin and 0.2% Triton X-100 for 1 h. Afterward, the cells were probed with monoclonal mouse anti-ICAM-1 antibody, monoclonal mouse anti-VCAM-1 antibody (dilutions, 1:100; Santa Cruz Biotechnology, Santa Cruz, CA, USA), or polyclonal rabbit antiphosphorylated IκBα (Ser32) antibody (dilution, 1:200; Cell Signaling Technology, Inc., Danvers, MA, USA) at 4°C for overnight. After being washed with PBS, the cells were incubated with fluorescein isothiocyanate (FITC)-conjugated secondary antibody, including anti-mouse immunoglobulin G (IgG) and anti-rabbit IgG (dilutions, 1:200; Jackson ImmunoResearch Laboratories, West Grove, PA, USA), at room temperature for 1 h. Negative control experiments were performed by incubating with PBS, instead of primary antibodies. The optical density of each well was determined at excitation and emission wavelengths of 485 and 520 nm using a SpectraMax microplate reader (Molecular Devices, Sunnyvale, CA, USA). Optical density of the negative control as background was subtracted from that of each well. Data were expressed as fold changes compared with the control.

### 2.8. Quantification of Monocyte Adhesion

A monocyte adhesion assay was carried out as we reported previously [9]. In brief, confluent HUVECs in 96-well plates (1 × 10^4^ cells/well) were incubated with Ang II for 6 h as already mentioned. Then, THP-1 cells were fluorescently labeled with 1 μmol/L carboxyfluorescein diacetate succinimidyl ester at 37°C for 30 min before addition of the monocytes to the HUVECs. Afterward, the fluorescence-labeled monocytes were cocultured with HUVECs and allowed to attach at 37°C for 30 min in a humidified atmosphere containing 5% CO_2_. After the removal of nonadherent monocytes, the residual cells were rinsed three to five times with PBS and incubated with PBS containing 2% Triton X-100 to lyse the attached monocytes. The total fluorescent intensity of cell lysates was determined by the use of a SpectraMax microplate reader (Molecular Devices, Sunnyvale, CA, USA) at excitation and emission wavelengths of 485 and 520 nm. In another set of experiments, HUVECs were cultured in 24-well plates at a density of 0.5 × 10^4^ cells/well. Fluorescence staining of monocyte adhesion to HUVECs was carried out as mentioned above. Succinctly, nonattached prelabeled monocytes were gently aspirated, and residual cells were rinsed three times with PBS. Finally, images were captured at excitation and emission wavelengths of 485 and 520 nm using a fluorescence microscope (DMI 3000 B, Leica, Wetzlar, Germany).

### 2.9. Measurement of Nuclear Factor Kappa B (NF-κB) Activity

HUVECs in 6-well plates (3 × 10^5^ cells/well) were incubated with AS-IV (30 μmol/L) or SNP (30 μmol/L) in the presence or absence of L-NMMA (1 mmol/L), LY294002 (10 μmol/L), or EGTA (500 nmol/L) for 1 h, followed by Ang II exposure for a further 30 min. After incubation with Ang II, a nuclear extraction kit (Active Motif, Carlsbad, CA, USA) was used to purify nuclear protein from the cells following the manufacturer's instruction. The NF-κB activity was determined using a nonradioactive, ELISA-based TransAM NF-κB p65 transcription factor assay kit (Active Motif, Carlsbad, CA, USA) as we described previously [9]. In brief, isolated nuclear proteins were added into each well coated with an unlabeled oligonucleotide containing the consensus binding site for NF-κB (5′-GGGACTTTCC-3′) and incubated for 1 h. After washing, a primary anti-NF-κB p65 subunit antibody was added and incubated for 1 h, followed by addition of a secondary horseradish peroxidase-conjugated antibody and incubation for 1 h. After washing was completed, a colorimetric reaction was initiated with addition of a developing solution prior to being terminated by a stop solution. The absorbance of each well was assayed at 450 nm using a SpectraMax microplate reader (Molecular Devices, Sunnyvale, CA, USA). The concentration of protein in the nuclear extract was determined using a Bio-Rad protein assay kit (Bio-Rad Laboratories, Hercules, CA, USA). The NF-κB activity was normalized to the protein concentration of the nuclear extract.

### 2.10. Statistical Analysis

All results were expressed as means ± standard error (SE) from at least four independent experiments. Comparisons between groups at each experimental time point were evaluated using two-way analysis of variance (ANOVA) followed by a Bonferroni's test. The differences among groups were analyzed by the use of one-way ANOVA with a Bonferroni's adjustment for multiple comparisons. A value of *p* < 0.05 indicated a statistically significant difference. All analyses were performed by the use of GraphPad Prism 6.0 software (GraphPad Software, Inc., La Jolla, CA, USA).

## 3. Results

### 3.1. AS-IV Had an Effect on the Viability of HUVECs

**Figure 1:**
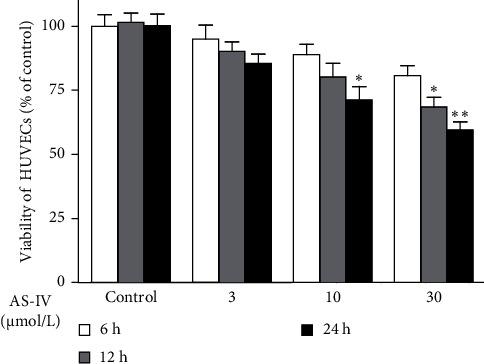
Effect of astragaloside IV (AS-IV) on the viability of human umbilical vein endothelial cells (HUVECs). HUVECs were incubated with AS-IV at a concentration of 3, 10, or 30 µmol/L for the indicated times. Cell viability was determined by a 3-(4,5-dimethylthiazol-2-yl)-2,5-diphenyltetrazolium bromide assay. *⁣*^*∗*^*p* < 0.05 and *⁣*^*∗∗*^*p* < 0.01 compared with the corresponding control group.

As demonstrated in Figure 1, compared with the control, the viability of HUVECs was decreased 12 or 24 h after incubation with AS-IV at a concentration of 10 or 30 μmol/L, respectively. However, no changes in the viability of HUVECs were found when HUVECs were incubated with AS-IV at a concentration of 3, 10, or 30 μmol/L for 6 h. Based on the data, 3–30 μmol/L AS-IV and an incubation duration of 6 h were chosen for the multiplex assay in the subsequent experiments.

### 3.2. AS-IV Increased NO Production Possibly via the Activation of Ca^2+^/PI3K/Akt/eNOS Pathway in HUVECs

**Figure 2:**
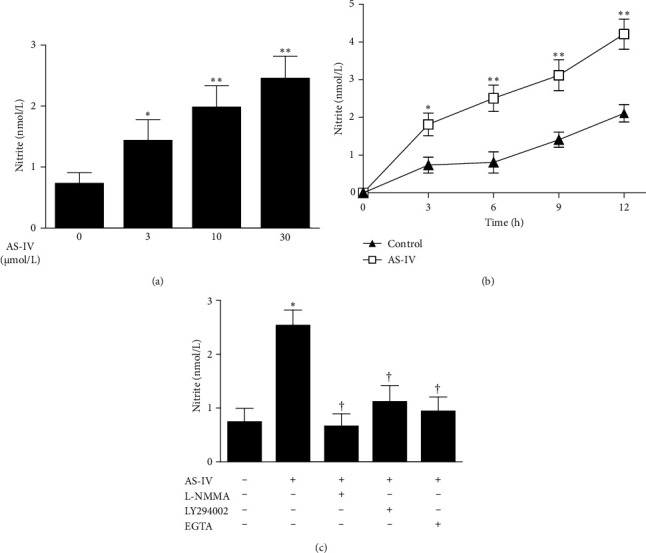
Effect of astragaloside IV (AS-IV) on nitric oxide production in endothelial cells: (A) human umbilical vein endothelial cells (HUVECs) were incubated with various concentrations of AS-IV (3, 10, or 30 µmol/L) for 6 h, (B) HUVECs were incubated with AS-IV (30 µmol/L) for 0–12 h, and (C) HUVECs were preincubated with or without N^G^-monomethyl-L-arginine (L-NMMA, 1 mmol/L), LY294002 (10 µmol/L), or ethylene glycol tetraacetic acid (EGTA, 500 nmol/L) for 1 h and then incubated with AS-IV (30 µmol/L) for an additional 6 h. The levels of nitrite in the culture supernatant were measured by the use of Griess assay. *⁣*^*∗*^*p* < 0.05 and *⁣*^*∗∗*^*p* < 0.01 compared with the control group; †*p* < 0.05 compared with HUVECs exposed to AS-IV alone.

We first examined the effect of AS-IV on NO production and its possible mechanisms in HUVECs. As demonstrated in Figure 2A,B, incubation with AS-IV (3, 10, or 30 μmol/L) for 6 h concentration-dependently enhanced the levels of nitrite in cultured medium, and the nitrite production induced by AS-IV (30 μmol/L) was time-dependently elevated. As shown in Figure 2C, AS-IV–mediated increase in nitrite production was prevented by preincubation with L-NMMA (a nonselective inhibitor of NOS, 1 mmol/L), which suggests that AS-IV–mediated NO production was specifically due to the activation of eNOS. Moreover, AS-IV–mediated increase in nitrite production was abolished by preincubation with LY294002 (a specific inhibitor of PI3K/Akt pathway, 10 μmol/L) or EGTA (500 nmol/L) that deprives extracellular Ca^2+^ from cultured medium. Taken together, these data suggest AS-IV triggers increase in NO production possibly via the activation of Ca^2+^/PI3K/Akt/eNOS pathway in endothelial cells.

### 3.3. AS-IV Increased the Expression of Phosphorylated eNOS^Ser1177^ Possibly via the Activation of Ca^2+^- and PI3K/Akt-Dependent Pathway in HUVECs

**Figure 3:**
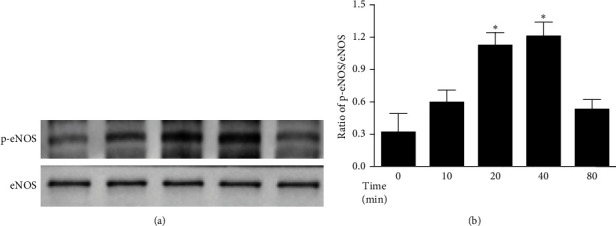
Effect of astragaloside IV (AS-IV) on the phosphorylation of endothelial nitric oxide synthase (p-eNOS) in endothelial cells. Human umbilical vein endothelial cells (HUVECs) were incubated with AS-IV (30 µmol/L) for the indicated times. Cell lysates were immunoblotted with the antibodies against p-eNOS at Ser1177 or eNOS. (A) Representative western blot of p-eNOS in HUVECs. (B) Bar graph shows the relative optical density values for p-eNOS in HUVECs. Data were expressed as the ratio of p-eNOS to the corresponding eNOS. *⁣*^*∗*^*p* < 0.05 compared with the control group.

**Figure 4:**
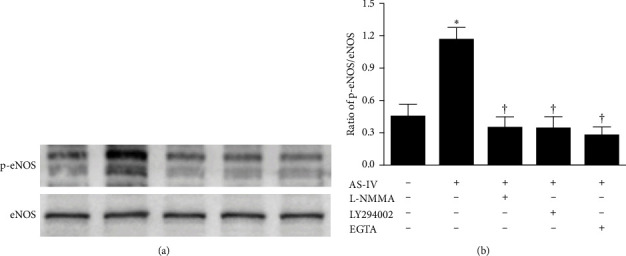
Effect of Ca^2+^- and phosphatidylinositol 3-kinase (PI3K)/serine/threonine kinase Akt-dependent signaling pathway on the astragaloside IV (AS-IV)-induced phosphorylation of endothelial nitric oxide synthase (p-eNOS) in endothelial cells. Human umbilical vein endothelial cells (HUVECs) were preincubated with or without N^G^-monomethyl-L-arginine (L-NMMA, 1 mmol/L), LY294002 (10 µmol/L), or ethylene glycol tetraacetic acid (EGTA, 500 nmol/L) for 1 h and then incubated with AS-IV (30 µmol/L) for an additional 40 min. Cell lysates were immunoblotted with the antibodies against p-eNOS at Ser1177 or eNOS. (A) Representative western blot of p-eNOS in HUVECs. (B) Bar graph shows the relative optical density values for p-eNOS in HUVECs. Data were expressed as the ratio of p-eNOS to corresponding eNOS. *⁣*^*∗*^*p* < 0.05 compared with the control group; †*p* < 0.05 compared with HUVECs exposed to AS-IV alone.

It has been well accepted that the phosphorylation of Ser1177 is implicated in the regulation of eNOS activity. As illustrated in Figures 3 and 4, the phosphorylation of eNOS at Ser1177 in HUVECs peaked at 40 min after incubation with AS-IV (30 μmol/L) and gradually decreased to the basal level thereafter. This response was abrogated by preincubation with L-NMMA (1 mmol/L), LY294002 (10 μmol/L), or EGTA (500 nmol/L) for 1 h. Consistent with the data on NO measurement, these results indicate that Ca^2+^- and PI3K/Akt-dependent signaling pathway may be essential for the AS-IV–induced phosphorylation of eNOS at Ser1177.

### 3.4. AS-IV Attenuated Ang II–Mediated Production of Proinflammatory Cytokines and Chemokines Possibly via the Activation of Ca^2+^/PI3K/Akt/eNOS/NO Pathway in HUVECs

**Figure 5:**
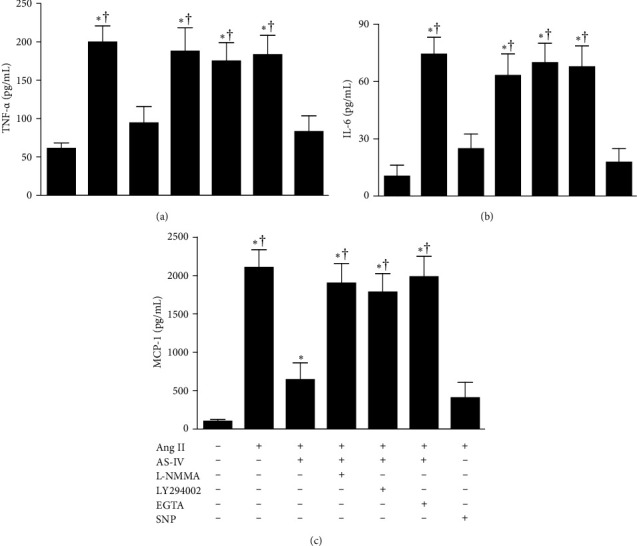
Effect of astragaloside IV (AS-IV) on angiotensin II (Ang II)-induced production of proinflammatory cytokines/chemokines including TNF-α (A), IL-6 (B), and MCP-1 (C) in endothelial cells. Human umbilical vein endothelial cells were preincubated with AS-IV (30 µmol/L) or sodium nitroprusside (SNP, 30 µmol/L) in the presence or absence of N^G^-monomethyl-L-arginine (L-NMMA, 1 mmol/L), LY294002 (10 µmol/L), or ethylene glycol tetraacetic acid (EGTA, 500 nmol/L) for 1 h and then incubated with Ang II (1 µmol/L) for an additional 6 h. The enzyme-linked immunosorbent assay (ELISA) assay was used to assay the concentrations of proinflammatory cytokines/chemokines in the culture medium. *⁣*^*∗*^*p* < 0.05 compared with the control group; †*p* < 0.05 compared with HUVECs exposed to Ang II in the presence of AS-IV.

Accumulating evidence shows that NO derived from eNOS plays an inhibitory role in the development of inflammation. Therefore, this study first examined whether AS-IV attenuates Ang II–mediated production of proinflammatory cytokines and chemokines via the activation of Ca^2+^/PI3K/Akt/eNOS/NO pathway in HUVECs. As shown in Figure 5, incubation with Ang II (1 μmol/L) for 6 h enhanced the production of proinflammatory cytokines and chemokines, including TNF-α, IL-6, and MCP-1, in HUVECs. The effects of Ang II were inhibited by concurrent incubation with AS-IV (30 μmol/L) or SNP (30 μmol/L). In addition, preincubation with L-NMMA (1 mmol/L), LY294002 (10 μmol/L), or EGTA (500 nmol/L) 1 h prior to incubation with AS-IV (30 μmol/L) completely abrogated the attenuating effects of AS-IV on Ang II–mediated production of inflammatory mediators. Incubation with L-NMMA, LY294002, or EGTA alone did not affect Ang II–induced production of inflammatory mediators (data not shown). Collectively, these results indicate that AS-IV inhibits Ang II–induced inflammation of endothelial cells possibly via the activation of Ca^2+^/PI3K/Akt/eNOS/NO pathway.

### 3.5. AS-IV Attenuated Ang II–Mediated Expression of Adhesion Molecules Possibly via the Activation of Ca^2+^/PI3K/Akt/eNOS/NO Pathway in HUVECs

**Figure 6:**
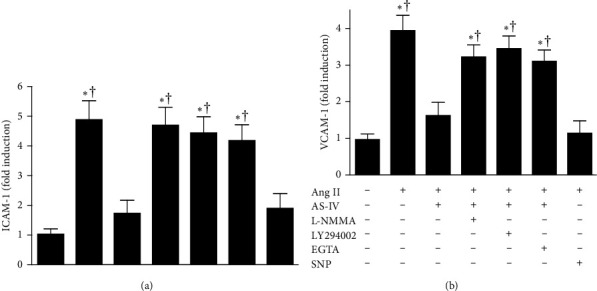
Effect of astragaloside IV (AS-IV) on angiotensin II (Ang II)-induced expression of ICAM-1 (A) and VCAM-1 (B) in endothelial cells. Human umbilical vein endothelial cells (HUVECs) were preincubated with AS-IV (30 µmol/L) or sodium nitroprusside (SNP, 30 µmol/L) in the presence or absence of N^G^-monomethyl-L-arginine (L-NMMA, 1 mmol/L), LY294002 (10 µmol/L), or ethylene glycol tetraacetic acid (EGTA, 500 nmol/L) for 1 h and then incubated with Ang II (1 µmol/L) for an additional 6 h. The cell-based enzyme-linked immunosorbent assay (ELISA) assay was used to quantity the expression of ICAM-1 and VCAM-1 in HUVECs. *⁣*^*∗*^*p* < 0.05 compared with the control group; †*p* < 0.05 compared with HUVECs exposed to Ang II in the presence of AS-IV.

Adhesion molecules have been demonstrated to play a critical role in the infiltration of leukocytes into numerous tissues. Thus, the present study subsequently examined whether AS-IV attenuates Ang II–mediated expression of adhesion molecules via the activation of Ca^2+^/PI3K/Akt/eNOS/NO signaling in HUVECs. As demonstrated in Figure 6, incubation with Ang II (1 μmol/L) for 6 h increased the expression of adhesion molecules, including ICAM-1 and VCAM-1. The Ang II–induced responses were attenuated by concurrent incubation with AS-IV (30 μmol/L) or SNP (30 μmol/L). Moreover, preincubation with AS-IV in the presence of L-NMMA (1 mmol/L), LY294002 (10 μmol/L), or EGTA (500 nmol/L) completely abolished its inhibitory effects on the expression of adhesion molecules. Incubation with L-NMMA, LY294002, or EGTA alone had no effect on Ang II–induced expression of adhesion molecules (data not shown). Together, these results suggest the diminished expression of adhesion molecules mediated by AS-IV may be attributable to the activation of Ca^2+^/PI3K/Akt/eNOS/NO pathway.

### 3.6. AS-IV Attenuated Ang II–Mediated Monocyte Attachment to Endothelial Cells Possibly via the Activation of Ca^2+^/PI3K/Akt/eNOS/NO Signaling Pathway

**Figure 7:**
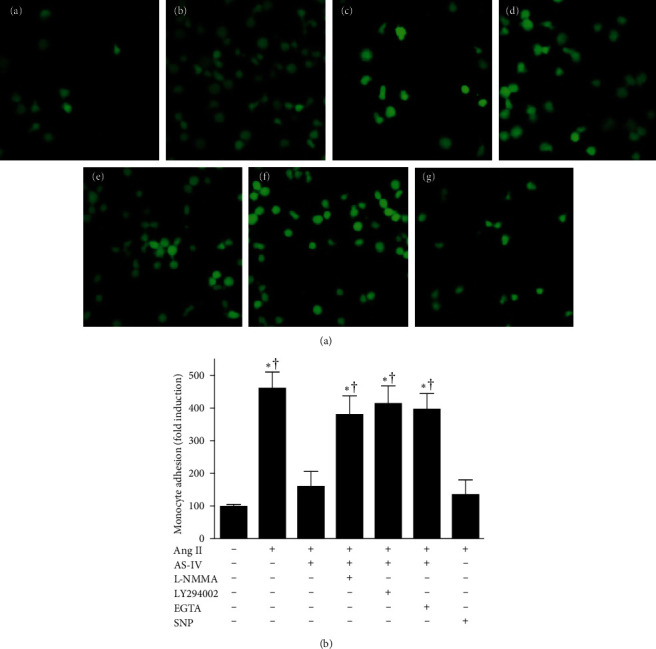
Effect of astragaloside IV (AS-IV) on angiotensin II (Ang II)-induced monocyte adhesion to endothelial cells. Human umbilical vein endothelial cells (HUVECs) were preincubated with AS-IV (30 µmol/L) or sodium nitroprusside (SNP, 30 µmol/L) in the presence or absence of N^G^-monomethyl-L-arginine (L-NMMA, 1 mmol/L), LY294002 (10 µmol/L), or ethylene glycol tetraacetic acid (EGTA, 500 nmol/L) for 1 h and then incubated with Ang II (1 µmol/L) for an additional 6 h and subsequently coincubated with fluorescence-stained human monocytes for 30 min. (A) Representative photomicrographs of fluorescence-labeled human monocyte adhesion to HUVECs. Magnification, ×200. HUVECs were incubated with vehicle (control) (a) or Ang II alone (b) and in the presence of AS-IV (c), L-NMMA (d), LY294002 (e), EGTA (f), or SNP (g) before coincubation with fluorescence-stained human monocytes. (B) Fluorescence intensity of human monocytes was determined by the use of a spectrofluorometer. *⁣*^*∗*^*p* < 0.05 compared with the control group; †*p* < 0.05 compared with HUVECs exposed to Ang II in the presence of AS-IV.

It is well established that adhesion of leukocytes to endothelial cells via adhesion molecules is a crucial step in the initiation of inflammation. Therefore, the current study investigated whether AS-IV inhibits attachment of monocytes to endothelial cells via the activation of Ca^2+^/PI3K/Akt/eNOS/NO signaling pathway. As shown in Figure 7, incubation with Ang II (1 μmol/L) for 6 h increased the number of monocytes that attached to HUVECs. Concurrent incubation with AS-IV (30 μmol/L) or SNP (30 μmol/L) suppressed Ang II–triggered monocyte adhesion. The attenuating effect of AS-IV was completely abolished by preincubation with L-NMMA (1 mmol/L), LY294002 (10 μmol/L), or EGTA (500 nmol/L) 1 h prior to incubation with AS-IV. Incubation with L-NMMA, LY294002, or EGTA alone did not affect Ang II–induced monocyte adhesion (data not shown). Overall, the data suggest that AS-IV can inhibit Ang II–induced monocyte attachment possibly via the activation of Ca^2+^/PI3K/Akt/eNOS/NO pathway.

### 3.7. AS-IV Attenuated Ang II–Mediated NF-κB Activation Possibly via the Activation of Ca^2+^/PI3K/Akt/eNOS/NO Signaling Pathway in HUVECs

**Figure 8:**
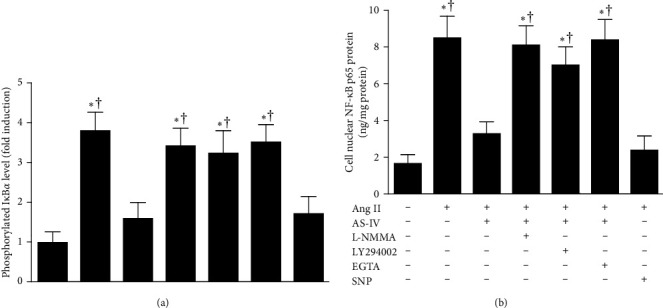
Effect of astragaloside IV (AS-IV) on angiotensin II (Ang II)-induced increases in the protein levels of phosphorylated IκBα (A) and activities of NF-κB (B) in endothelial cells. Human umbilical vein endothelial cells (HUVECs) were preincubated with AS-IV (30 µmol/L) or sodium nitroprusside (SNP, 30 µmol/L) in the presence or absence of N^G^-monomethyl-L-arginine (L-NMMA, 1 mmol/L), LY294002 (10 µmol/L), or ethylene glycol tetraacetic acid (EGTA, 500 nmol/L) for 1 h and then incubated with Ang II (1 µmol/L) for an additional 30 min. The protein levels of phosphorylated IκBα and activities of NF-κB in HUVECs were measured using a cell-based enzyme-linked immunosorbent assay (ELISA) and a specific TransAM NF-κB p65 Transcription Factor Assay Kit, respectively. *⁣*^*∗*^*p* < 0.05 compared with the control group; †*p* < 0.05 compared with HUVECs exposed to Ang II in the presence of AS-IV.

It has been recognized that NF-κB is a critical transcriptional factor for the expression of inflammatory mediators. Since phosphorylation of IκBα and subsequent nuclear translocation of NF-κB are required for the activation of NF-κB, this study determined the inhibitory effect of AS-IV on Ang II–triggered NF-κB activation by assaying the phosphorylation of IκBα at Ser32 and NF-κB activity. As illustrated in Figure 8, the phosphorylation of IκBα at Ser32 and NF-κB activity elevated 30 min after exposure to Ang II (1 μmol/L) in HUVECs, and these responses were inhibited by concurrent incubation with AS-IV (30 μmol/L) or SNP (30 μmol/L). Moreover, the inhibitory effect of AS-IV was abolished by preincubation with L-NMMA (1 mmol/L), LY294002 (10 μmol/L), or EGTA (500 nmol/L) 1 h before incubation with AS-IV. Incubation with L-NMMA, LY294002, or EGTA alone had no effect on Ang II–induced increases in the protein levels of phosphorylated IκBα and activities of NF-κB (data not shown). Taken together, these results indicate that AS-IV suppresses Ang II–induced NF-κB activation possibly via the activation of Ca^2+^/PI3K/Akt/eNOS/NO pathway.

## 4. Discussion

The major finding of our study is that AS-IV–mediated NO production attenuates Ang II–induced inflammatory response via the inhibition of NF-κB activity in endothelial cells, which is featured by a reduction in proinflammatory cytokine/chemokine production and adhesion molecule expression, and subsequent adhesion of monocytes to endothelial cells. This reaction may be dependent on the activation of Ca^2+^ and PI3K/Akt/eNOS/NO signaling cascade. Collectively, the results indicate that AS-IV inhibits Ang II–mediated inflammatory response of endothelial cells possibly via the activation of Ca^2+^ and PI3K/Akt/eNOS/NO pathway.

It is well recognized that plant-derived products are attractive sources of novel bioactive components for medicines, and they are effective to treat various cardiovascular diseases and improve the quality of life of patients with long-lasting diseases [19]. *Astragalus membranaceus Bunge* is one of the most frequently used traditional Chinese medicine since ancient times. Traditionally, it has been used to treat numerous diseases, including cardiovascular disorder and kidney and liver diseases [11, 12]. AS-IV is a saponin extracted from *Astragalus membranaceus Bunge*, and it has a wide range of pharmacological properties, including anti-inflammation, antioxidant stress, and myocardial protection [11, 12]. AS-IV was evaluated herein for its capacity to inhibit Ang II–induced inflammation in vitro. Our findings showed that AS-IV had anti-inflammatory effects since it attenuated the activation of NF-κB induced by Ang II and its inflammatory consequences. These effects may be dependent on Ca^2+^ and PI3K/Akt/eNOS/NO pathway.

The vascular endothelium is thought to protect against vascular pathology occurring in almost all types of cardiovascular disease [2, 3, 20]. Endothelial cells contribute to maintaining cardiovascular homeostasis, which includes tonus modulation, anti-inflammation, and anticoagulation [4, 21]. However, there is accumulating evidence showing that the disruption of endothelial cell homeostasis, termed endothelial dysfunction, is the predominant driving force for the development of various inflammation-characterized diseases such as atherosclerosis, hypertension, and ischemia–reperfusion injury [1, 2]. In the context of the diseases, endothelial cells are implicated in elevated cytokine/chemokine production as well as expression of adhesion molecules, thereby resulting in the attachment of leukocytes to the endothelium [3, 22].

Although RAS, an endocrine system, plays a substantial role in the regulation of blood pressure and volume under physiological conditions, systemic and local overreactivity of this system is obviously involved in the pathogenesis of cardiovascular diseases [6, 23]. Ang II is the principal bioactive component of the RAS; it mediates many of its pathological actions via triggering endothelial dysfunction in endothelial cells. Several lines of evidence have shown that Ang II is involved in endothelial dysfunction via promoting mitogen-activated protein kinases (MAPKs) and its prooxidant effects [6, 24]. Inflammation is one of the major accompanied consequences derived from endothelial dysfunction [4]. Many researches focused on the protection of endothelial cells against inflammation-induced injury have demonstrated an obvious diminution of tissue injury and supplied further evidence supporting a critical role played by endothelial cells in the pathogenesis of inflammation [25–27]. As previously reported [6], our results showed that inflammatory responses have markedly enhanced in HUVECs exposed to Ang II. Moreover, we also found that AS-IV inhibited Ang II–induced inflammation in HUVECs. Collectively, our data support the idea that AS-IV is a potential new intervention for the prevention and treatment of Ang II–triggered inflammation.

In mammals, there are three isoforms of NOS, including neuronal NOS (nNOS), eNOS, and inducible NOS (iNOS) [28]. In addition to its modulation of cardiovascular functions, NO contributes to the regulation of inflammatory responses. Low concentrations of NO formed by eNOS facilitate the production of the secondary messenger cyclic guanosine monophosphate (cGMP), which in turn mediates the actions of NO, including vasorelaxation, anti-inflammation, and anticoagulation [28, 29]. In this study, we found that AS-IV facilitated the release of NO from endothelial cells. Moreover, we also found that the AS-IV–mediated inhibition of inflammation was abolished by L-NMMA, a specific inhibitor of NOS. The results suggest that the NO production derived from eNOS contributes to the AS-IV–mediated inhibition of endothelial cell inflammation induced by Ang II. Indeed, considerable evidence shows that NO derived from eNOS is a potent inhibitor of inflammation in vivo and in vitro [9, 30].

NO produced by eNOS is considered to have a variety of properties. It is widely held that multiple mechanisms are involved to regulate the NO production mediated by eNOS. As already shown [31], the level of intracellular Ca^2+^ is a critical regulator, which prompts calmodulin to bind to calmodulin-binding domain of eNOS, leading to the activation of eNOS. Cellular Ca^2+^ is an important signaling molecule regulating multiple cellular functions. Although an elevation of cytosolic Ca^2+^ has been demonstrated to trigger inflammation [32], our results showed that the attenuating effects of AS-IV on Ang II–mediated increases in inflammatory responses were completely abrogated by preincubation with EGTA, a Ca^2+^-chelating agent. It is possible that EGTA induces a reduction in cytosolic Ca^2+^ and inhibits the activation of eNOS, thereby leading to an increase in inflammatory responses. The cascade of PI3K and its downstream Akt is another crucial factor controlling the activity of eNOS, which directly increases the phosphorylation of Ser1177 on eNOS, resulting in enhancing the sensitivity of eNOS to Ca^2+^/calmodulin [10, 19, 31]. Recently, accumulating evidence shows that many natural compounds, including AS-IV, have protective effects against cardiovascular and lung damage via the regulation of PI3K/Akt- and AMP-activated protein kinase (AMPK)-mediated signaling pathway [33–35]. In this study, our results demonstrated that the effects of AS-IV are linked to the PI3K/Akt/eNOS/NO pathway using pharmacological and molecular biological approaches. In agreement with previous studies [6], we found that inflammatory responses have markedly increased in HUVECs exposed to Ang II. Moreover, we also observed that the attenuation of PI3K/Akt/eNOS/NO pathway by LY294002, L-NMMA, or EGTA effectively abrogated the AS-IV–mediated inhibition of inflammation induced by Ang II in HUVECs. Collectively, the results suggest the possible involvement of the Ca^2+^/PI3K/Akt/eNOS/NO cascade in AS-IV–mediated anti-inflammatory actions in endothelial cells.

NF-κB is a crucial transcription factor, as it activates the expression of several inflammatory genes after inflammatory stimulation. Usually, NF-κB is retained in the cytoplasm bound with the IκBα. Inflammatory stimuli, such as lipopolysaccharide and Ang II, directly cause the activation of IκB kinase (IKK) complex, leading to phosphorylation and subsequent degradation of IκBα. Thus, NF-κB p65 is released and is subsequently translocated into the nucleus where it binds to the corresponding DNA sequences, thereby leading to the expression of inflammatory molecules [36]. It is well accepted that NO has anti-inflammatory properties. There is ample evidence demonstrating that NO inhibits NF-κB activity via induction and stabilization of IκBα [37, 38]. In addition, there are several studies showing that NO directly suppresses DNA binding activity of NF-κB through S-nitrosylation of certain cysteine residues [39, 40].

Usually, the toxicities of many traditional Chinese herbs and their extracts limit their application in clinical practices. No cytotoxic effects of AS-IV on HUVECs following 6 h exposure to higher concentrations were found in MTT assay. The results suggest normal cells appeared to be able to tolerate the agent at higher concentrations and support the safety of AS-IV for clinical applications. Based on the data, we speculate that AS-IV may be a valuable agent for the treatment of inflammatory diseases. However, further studies, including animal studies and clinical trials, are required to determine its safety in clinical applications.

Overall, our data showed that AS-IV was efficient in inhibiting Ang II–induced inflammatory responses in endothelial cells. The anti-inflammatory actions of AS-IV are mediated likely by the activation of Ca^2+^ and PI3K/Akt/eNOS/NO signaling pathway. The results imply that AS-IV may be an effective candidate for the treatment of Ang II–triggered inflammation.

## 5. Perspectives

There is considerable evidence suggesting that AS-IV is an attractive agent for the treatment of inflammation-related diseases, such as atherosclerosis and myocardial infarction [11, 12]. In the pathophysiology of inflammation, the actions by leukocytes are preceded by the production of inflammatory mediators in inflamed endothelial cells. This results in the infiltration of circulating leukocytes, which are necessary for inflammatory responses. Thus, clarifying the mechanisms by which AS-IV suppresses the inflammatory response of endothelial cells is important for considering it as a valuable new candidate to treat inflammation. In this regard, our study sheds light on how AS-IV inhibits Ang II–induced inflammatory responses in endothelial cells and supports the idea that AS-IV is a potential new candidate for the prevention and treatment of Ang II–mediated inflammation.

## Data Availability

All data used and/or analyzed during the current study are included in the article.
